# CCDC134 facilitates T cell activation through the regulation of early T cell receptor signaling

**DOI:** 10.3389/fimmu.2023.1133111

**Published:** 2023-05-10

**Authors:** Tianzhuo Zhang, Qianwen Shi, Huining Gu, Biaoyi Yu, Sha Yin, Qing Ge, Xiaoning Mo, Xiaofeng Liu, Jing Huang

**Affiliations:** ^1^ Department of Immunology, School of Basic Medical Sciences, Peking University, and National Health Commission (NHC) Key Laboratory of Medical Immunology, Peking University, Beijing, China; ^2^ Shaanxi Institute for Pediatric Diseases, Xi’an Key Laboratory of Children’s Health and Diseases, Xi’an Children’s Hospital, The Affiliated Children’s Hospital of Xi’an Jiaotong University, Xi’an, Shaanxi, China; ^3^ Hepatopancreatobiliary Surgery Department I, Key laboratory of Carcinogenesis and Translational Research, Ministry of Education/Beijing, Peking University Cancer Hospital & Institute, Beijing, China

**Keywords:** CCDC134, T cell activation, T cell receptor signaling, CD3ϵ, ubiquitination

## Abstract

Modulation of surface T cell antigen receptor (TCR) expression is crucial for proper T cell development and maintenance of mature T cell function at steady state and upon stimulation. We previously determined that CCDC134 (coiled-coil domain containing 134), a cytokine-like molecule that served as a potential member of the γc cytokine family, contributes to antitumor responses by augmenting CD8^+^ T cell-mediated immunity. Here we show that T cell-specific deletion of *Ccdc134* decreased peripheral mature CD4^+^ and CD8^+^ T cells, which resulted in impaired T cell homeostasis. Moreover, *Ccdc134*-deficient T cells exhibited an attenuated response to TCR stimulation *in vitro*, showing lower activation and proliferative capacity. This was further reflected *in vivo*, rendering mice refractory to T cell-mediated inflammatory and antitumor responses. More importantly, CCDC134 is associated with TCR signaling components, including CD3ϵ, and attenuated TCR signaling in *Ccdc134-*deficient T cells *via* altered CD3ϵ ubiquitination and degradation. Taken together, these findings suggest a role for CCDC134 as a positive regulator of TCR-proximal signaling and provide insight into the cell-intrinsic functional consequences of *Ccdc134* deficiency in the attenuation of T cell-mediated inflammatory and antitumor responses.

## Introduction

T cells are crucial players in adaptive immune responses that control inflammatory disorders and the development of neoplastic lesions (1). Upon antigen stimulation, naïve T cells are activated and subsequently induced to proliferate and differentiate into effector subsets that mediate various immune functions (2). Activation of naïve T cells is initiated by the interaction of TCR-CD3 complexes on T cells with cognate peptide/MHC complexes on antigen-presenting cells (APC), and requires ligation of additional co-stimulatory molecules such as CD28. Co-stimulation of TCR-CD28 triggers signaling cascades that regulate both the initial activation of naïve T cells and their subsequent differentiation.

The TCR-CD3 complex is a multimeric receptor composed of four noncovalent dimers: the antigen-binding TCRαβ or γδ heterodimer, and the CD3γϵ, CD3δϵ, and CD3ζζ dimers (3). TCR-CD3 subunits undergo a finely regulated process of assembly and secretion *via* the endoplasmic reticulum (ER) and the Golgi apparatus to ensure expression of the complex on the cell surface. Cell surface TCR-CD3 complex levels represent a balance between internalization, recycling, and degradation of existing complexes and the expression of new ones that must be assembled and transported to the cell surface (4). Moreover, dynamic regulation of cell surface TCR-CD3 complex levels through multiple pathways ensures appropriate signals during T cell development and can modulate T cell effector functions. Upon antigen stimulation, the TCR-CD3 complex on mature T cells is rapidly downmodulated through enhanced internalization and degradation, as well as reduced recycling, thereby avoiding T cell hyperactivation and autoimmunity (5).

Degradation of TCR-CD3 complex subunits is a control mechanism that operates at many stages of the TCR life cycle. During complex assembly, unassembled proteins are retained in the ER and eventually degraded by the lysosome-dependent or -independent process. TCRα and TCRβ complexes are degraded after ER retention by cytosolic proteasomal degradation mediated by ubiquitinated intermediates (6). CD3ζ, a major component of the TCR-CD3 complex that is essential for its function, is located in vesicles distinct from the ER that can be translocated to the plasma membrane. Other TCR chains, such as CD3ϵ and CD3γ, are primarily retained in the ER and traffic toward the plasma membrane through the constitutive secretory pathway and are individually expressed *via* ubiquitin intermediates (7–9). TCR engagement by antigen also leads to TCR-CD3 internalization and ubiquitin-mediated degradation (10) to limit T cell activation. Degradation of CD3ζ chains occurs in lysosomes and depends on the ubiquitination of some of the subunits, while TCRα and CD3δϵ are degraded from the ER in a proteasome-dependent manner (11).

TCR signaling is initiated by activation of the protein tyrosine kinase Lck, which phosphorylates immunoreceptor tyrosine-based activation motifs (ITAMs) on CD3ζ triggering recruitment of the tyrosine kinase ZAP70 to doubly phosphorylated CD3ζ (12). Lck can also phosphorylate ZAP70, stabilizing its active conformation and upregulating its catalytic activity (13). In turn, active ZAP70 phosphorylates the transmembrane adaptor LAT, which recruits the scaffold protein SLP76 *via* the adaptor PLCγ1 and transduces the TCR signal to various downstream pathways that includes activation of MAP kinases, IκB kinase and several families of transcription factors. These signaling events induce T cell expansion and production of cytokines such as IL-2 and IFN-γ (14). Therefore, the strength of the TCR signal play a crucial role on the nature and magnitude of an immune response. Although numerous molecular components that regulate TCR signaling have been characterized, the interactions between these molecules and their assembly into complexes have remained elusive.

Coiled-coil domain containing 134 (CCDC134) is a secreted protein that has been reported to inhibit MAPK phosphorylation, and is critical for mouse embryonic development (15, 16). CCDC134 might serve as a cytokine-like molecule that exerts antitumor effects by promoting activation and proliferation of CD8^+^ T cells in exocrine form (17). It has also been shown to exert potent anti-inflammatory effects through selective modulation of Th1 and Th17 cells (18). Despite well-documented antitumor and anti-inflammatory properties, the molecular mechanism of CCDC134 as a multifaceted scaffolding protein to regulate T cells remains unclear. In the current study we generated and analyzed T cell-specific *Ccdc134*-deficient mice. We found that *Ccdc134* deficiency resulted in fewer peripheral CD4^+^ and CD8^+^ T cells and impaired early T cell activation by promoting CD3ϵ ubiquitination. This rendered mice refractory to T cell-mediated inflammatory and antitumor responses. Our data suggest a role for CCDC134 as a critical component of the TCR signalosome that modulates T cell activation through the regulation of TCR signaling.

## Materials and methods

### Mice

*Ccdc134^fl/fl^* mice on a C57BL/6N background were described previously (16). Homozygous *Ccdc134* ^fl/fl^ mice were crossed with *Cd4*-Cre or *Lck*-Cre transgenic mice to produce age-matched *CD4-cre^–^Ccdc134 ^fl/fl^* or *Lck-cre^–^Ccdc134 ^fl/fl^* as WT control, and T cell-conditional *Ccdc134* knockout mice, including *CD4-cre^+^Ccdc134 ^fl/fl^* or *Lck-cre^+^Ccdc134 ^fl/fl^* as TKO. Experiments were performed with young adult (6- to 8-week-old) female and male mice. All mice were on the C57BL/6 genetic background and maintained in a specific-pathogen-free facility of Peking University Health Science. All experimental procedures were approved and performed according to the Guidelines for the Care and Use of Animals (Animal Care Committee of Peking University Health Science, Beijing, China) (LA2016153).

### Cell lines, antibodies, and reagents

HEK293T, B16F10 (High Metastatic Mouse Melanoma), and LLC1 (Lewis Lung Carcinoma) cell lines were from ATCC between 2002 and 2003. Functional-grade anti-mouse CD3ϵ (145-2C11) and CD28 (37.51) were from BD Bioscience. Antibodies for phospho-Lck (Tyr505), phospho-SLP76 (Ser376), phospho-Zap70 (Tyr329)/Syk (Tyr352), phospho-PLCγ1 (Tyr783), phospho-LAT (Tyr220), Lck, LAT (E3U6), CD3ϵ (CD3-12) and anti-Flag (M2) were from Cell Signaling Technology. The rabbit anti-CCDC134 (ab106442) antibody was from Abcam Biotechnology (Cambridge, MA). Biotin anti-mouse CD3ϵ and biotin anti-mouse CD71 (transferrin receptor, TfR) were from Biolegend. Anti-His, and anti-HA antibodies were from Proteintech, and anti-β-actin (C-4) was from ZSGB-BIO.

Fluorescently labeled anti-mouse CD4 (L3T4), CD8 (53-6.7), CD3 (145-2C11), TCRβ (H57-597), CD44 (IM7), CD62L (MEL-14), CD45 (30-F11), CD127 (A7R34), KLRG1 (2F1), PD1 (29F.1A12), Tim3 (B8.2C12) and IFN-γ (XMG1.2) antibodies were purchased from Biolegend. Recombinant mouse IL-2 was from Peprotech. The reagents for mouse IL-2, TNF-α, and IFN-γ ELISAs were from eBioscience.

### Isolation of murine splenocytes and cell cultures

Splenic single-cell suspension was prepared by mechanical disruption followed by red blood cell lysis for 10 min at room temperature, then centrifuged and cultured at 10^7^ cells/ml. Murine splenocytes and isolated primary mouse T cells from WT or *Ccdc134* TKO mice were stimulated *in vitro* using plate-bound anti-CD3ϵ (2 μg/mL) and soluble anti-CD28 (1 μg/mL). All primary T cells and HEK293T cells were cultured in RPMI media supplemented with 10% FCS, 2 mM L-glutamine, and penicillin/streptomycin and maintained at 37°C in 5% CO_2_. All cell lines were tested for mycoplasma contamination and authenticated utilizing short tandem repeat profiling. The same batch of cells was thawed every month.

### Flow cytometric analysis and cell sorting.

Splenocyte and lymph node single-cell suspensions were subjected to flow cytometric analysis using FACS Canto (BD Biosciences) and cell sorting using FACS AriaII (BD Biosciences). For intracellular cytokine staining, T cells isolated from spleens, draining lymph nodes (LN), or tumors were stimulated for 4 h with PMA (50 ng/mL) and ionomycin (500 ng/mL) in the presence of BFA (10 μg/mL). The cells were stained with surface markers, fixed with 4% formaldehyde for 20 minutes, permeabilized with 0.5% saponin, and subjected to intracellular cytokine staining and flow cytometry.

Apoptosis was detected by incubating cells in binding buffer (10 mM HEPES pH 7.4, 140 mM NaCl, 2.5 mM CaCl_2_) with Annexin-V-FITC and 7-Aminoactinomycin D (7AAD). For cell proliferation assays, T cells were resuspended at 0.5–50×10^6^ cells/mL in 5% FBS/PBS and labeled with 2.5 mmol/L carboxyfluorescein succinimidyl ester (CFSE, Invitrogen) at 37°C for 5 minutes. CFSE-labeled cells were washed twice, and incubated in RPMI-1640 with 10% FBS. Cell division was detected by flow cytometry. FACS data were analyzed using FlowJo v.10 software.

### Immunofluorescence

Primary CD8^+^ T cells on cover glasses were washed with ice-cold PBS and fixed with acetone for 5 min. After blocking with PBS with 2% FBS for 30 min, the cells were incubated with rat anti-CD3ϵ antibody (CD3-12) and rabbit anti-CCDC134 antibody (ab106442) overnight at 4°C, and then incubated with TRITC-labeled anti-rat and FITC-conjugated anti-rabbit secondary antibodies for 30 min at 4°C. Samples were then washed and mounted in antifade reagent containing 2-(4-Amidinophenyl)-6-indolecarbamidine dihydrochloride (DAPI) for nuclei staining. After washing, images were observed and acquired using Leica TCS-SP8 STED 3X confocal microscope.

### TCR internalization assay

Murine CD4^+^ or CD8^+^ T cells (2×10^6^ cells) were treated with biotinylated anti-CD3ϵ (10 μg/ml) and non-biotinylated anti-CD28 (2 μg/ml) for 30 min on ice to bind and label all the cell surface CD3ϵ. Cells were washed to remove unbound antibodies, incubated at 37°C for the indicated times, and transferred to 4°C to terminate receptor internalization. The remaining surface CD3 was quantified by FACS analysis using APC-conjugated streptavidin. The % of TCR downregulation was based on the mean fluorescence intensity (MFI) of surface CD3 expression calculated as 100 × [(MFI at time (0) - MFI at time (t)]/MFI at time (0).

For measurement of the proteasome activity, the isolated primary mouse CD8^+^ T cells were treated with antibodies for 12 h, and then were pretreated with the proteasome inhibitor MG132 (20 μM) for 6 h before harvesting the cells. Afterward, the cell surface CD3ϵ and TCRβ were detected by FACS analysis.

### Immunoblot, coimmunoprecipitation, and ubiquitination assays

Sorted CD4^+^ or CD8^+^ T cells from spleens of WT or TKO mice were stimulated with plate-bound anti-CD3ϵ (2 μg/mL) and soluble anti-CD28 (1 μg/mL) at 37°C for the indicated times. Whole-cell lysates were extracted with RIPA lysis buffer (50 mM Tris–HCl, 150 mM NaCl, 1% NP-40, 0.5% sodium deoxycholate, and 1 mM EDTA, pH 7.4) containing protease and phosphatase inhibitors (complete Mini and PhosSTOP, Roche) and subjected to immunoblot and coimmunoprecipitation.

For immunoblot analysis of protein phosphorylation, the cell lysates were resolved on 5%–20% gradient gels and transferred to nitrocellulose membranes (Amersham Pharmacia, UK). Membranes were blocked with 5% nonfat milk and incubated with primary antibodies followed by HRP-conjugated secondary antibodies. The bands were visualized using enhanced chemiluminescence substrate system (PerkinElmer), followed by film exposure or digital imaging with an Amersham Imager 600 (GE Healthcare Life Sciences).

For coimmunoprecipitation assay, 1 mg of cell lysate was incubated with 2 µg of the indicated antibodies for 4 h or overnight at 4°C, and then crossed linked to 20 µL of Protein G Dynabeads (Thermo Fisher Scientific) and incubated for 1 h at 4°C. The beads were washed six times in lysis buffer and immunoprecipitates were eluted by heating the beads for 10 min at 95°C in 20 µL of SDS sample buffer containing 50 mM DTT followed by immunoblot analysis.

For protein degradation assay, 80–90% confluent cultures of HEK293T cells were transiently cotransfected with different plasmids, and incubated with cycloheximide (CHX, 15 μg/mL) 24 h later. Cells were harvested at the indicated time points and subjected to immunoblot analysis.

For *in vivo* ubiquitination assay, transiently transfected HEK293T cells and isolated primary mouse CD3^+^ T cells were treated with the proteasome inhibitor MG132 (20 μM) for 6 h, and then were lysed in RIPA buffer supplemented with 6 M urea and 4 mM N-ethylmaleimide. Lysates were subjected to CD3ϵ immunoprecipitation followed by immunoblot detection of ubiquitinated CD3ϵ.

### Delay-type hypersensitivity assay

Mice were sensitized in both hind footpads with 5 mg/mL methylated bovine serum albumin (mBSA, Sigma) in complete Freund’s adjuvant (CFA, Sigma). Seven days after sensitization, mice were challenged with 30 μL of 5 mg/mL of mBSA into one footpad and an equal volume of PBS into the other footpad. Footpad thickness was measured by caliper and footpad swelling was calculated as (cm) = [footpad thickness of mBSA-injected footpad (cm)] - [footpad thickness of PBS-injected footpad (cm)] as previously described (19). Meanwhile, whole lymph node cells (2×10^5^ cells) were prepared from inguinal draining lymph nodes and stimulated with anti-CD3 and anti-CD28 antibodies at 37°C for indicated time points. For cytokine production, cells were cultured for 2 days and supernatants were harvested and subjected to ELISA to determine IL-2 and IFN-γ concentrations (eBioscience).

### RNA sequencing analysis

Total CD8^+^ T cells from the spleens of 8-week-old WT and *Ccdc134* TKO mice were either immediately lysed for RNA preparation or activated for 24 h with anti-CD3 (2 μg/ml) and anti-CD28 (1 μg/ml). Total RNA was extracted using TRIzol (Invitrogen, Waltham, MA) and subjected to RNA sequencing analysis using an Illumina sequencer (NovelBio Bio-Pharm Technology Co., Ltd., Shanghai, China). The raw reads were then aligned to the reference genome (build mm10) using Tophat2 RNA-seq alignment software. The mapping rate was 70% overall across all samples in the dataset. The gene expression counts were quantified in HTseq-Count, and differential expression was analyzed using the R package DESeq2. P values obtained from multiple binomial tests were adjusted using the false discovery rate. The differentially expressed genes are defined by a Benjamini–Hochberg-corrected *p*-value <0.05, and subjected to gene-set enrichment analysis using gene ontology biological processes database (https://david.ncifcrf.gov/home.jsp). RNA sequencing data were analyzed by multiplot (http://www.bioinformatics.com.cn/).

### Tumor models

Age- and sex-matched WT and *Ccdc134* TKO mice were injected subcutaneously with 2×10^5^ B16-OVA cells, which is an ovalbumin-transfected clone derived from the murine melanoma cell line B16. Tumors were measured every other day and tumor volumes were calculated as (width^2^ × length)/2. Mice were considered to have a lethal disease when their tumor volume reached 2 cm^3^. Draining lymph nodes and tumors were isolated at the indicated time points. The tumors were homogenized through 200 mesh strainers to obtain single-cell suspensions. Red blood cells were lysed using an ammonium chloride lysis buffer, and tumor infiltrating lymphocytes (TILs) were isolated using Percoll (Solarbio). The collected cells were stained with different antibodies for flow cytometric analysis of immune cells.

### Identification of CCDC134-binding proteins by affinity purification and liquid chromatography-tandem mass spectrometry (LC-MS/MS)

CD8^+^ T cells isolated from spleens of WT and *Ccdc134* TKO mice were stimulated with anti-CD3 and anti-CD28 antibodies for 16 h. Whole cell lysates were extracted directly for LC-mass spectrometry (MS)/MS analysis.

Splenic cells from WT mice were stimulated with anti-CD3 and anti-CD28 antibodies for 24 h and cell lysates were harvested. In brief, 5 mg of precleared lysate was incubated with purified eukaryotic His-tagged mCCDC134, hCCDC134, or control protein (His-tagged human Igκ) at 4°C for 2 h, followed by 30 μL of anti-His conjugated magnetic beads at 4°C for 2 h. The resins were washed six times with a buffer containing 50 mM Tris-HCl, pH 7.5, 150 mM NaCl, 0.5% NP-40, and 1 mM EDTA. The elutes were extracted in loading sample buffer containing 50 mM DTT for 10 min at 95°C and resolved by SDS-PAGE followed by Coomassie blue staining. Three regions of the gel where the staining pattern of the experimental groups differed from the control were excised and subjected to in-gel trypsin digestion, and then were analyzed by LC-MS/MS.

MS/MS-based peptide and protein identifications were validated by Scaffold version 4.8.6 (Proteome Software). Proteins identified in both the experimental and control samples were regarded as nonspecific binding or contamination during sample preparation. Functional annotation and enrichment analysis of the GO terms associated with the identified mCCDC134- and hCCDC134-binding proteins were performed using the Database for Annotation, Visualization, and Integrated Discovery (DAVID) program (version 6.8). The differential proteins were analyzed by multiplot (http://www.bioinformatics.com.cn/).

### Statistical analysis

All data are presented as the mean ± SEM and analyzed using Prism GraphPad software v10.0. Statistical analysis of two experimental groups was performed using two-tailed unpaired Student’s t-tests. Comparisons of two groups with different time points were evaluated using two-way ANOVA with Geisser–Greenhouse correction followed by Dunn-Sidak *post hoc* tests. P values less than 0.05 were considered significant and the level of significance is indicated as * *p* < 0.05, ** *p* < 0.01, *** *p* < 0.001, **** *p* < 0.0001.

## Results

### T cell-specific *Ccdc134* deficiency impairs peripheral T cell homeostasis

Complete loss of *Ccdc134* on the C57BL/6 background results in embryonic lethality at embryonic day (E)12. To clarify the function of CCDC134 in T cell development, we crossed *Ccdc134^fl/fl^* mice with CD4 or proximal lymphocyte-specific protein tyrosine kinase (Lck) promoter-driven Cre transgenic mice (CD4-Cre or Lck-Cre) to generate *Ccdc134* conditional knockout mice with T cell-specific deletion (TKO). Genotyping, RT-PCR, and immunoblotting analysis all demonstrated specific deletion of *Ccdc134* in CD4^+^ and CD8^+^ (P) but not in CD4^–^CD8^–^ (N) splenocytes from WT and *Ccdc134* TKO mice (**Supplemental Figures 1A–C**).

**Figure 1:**
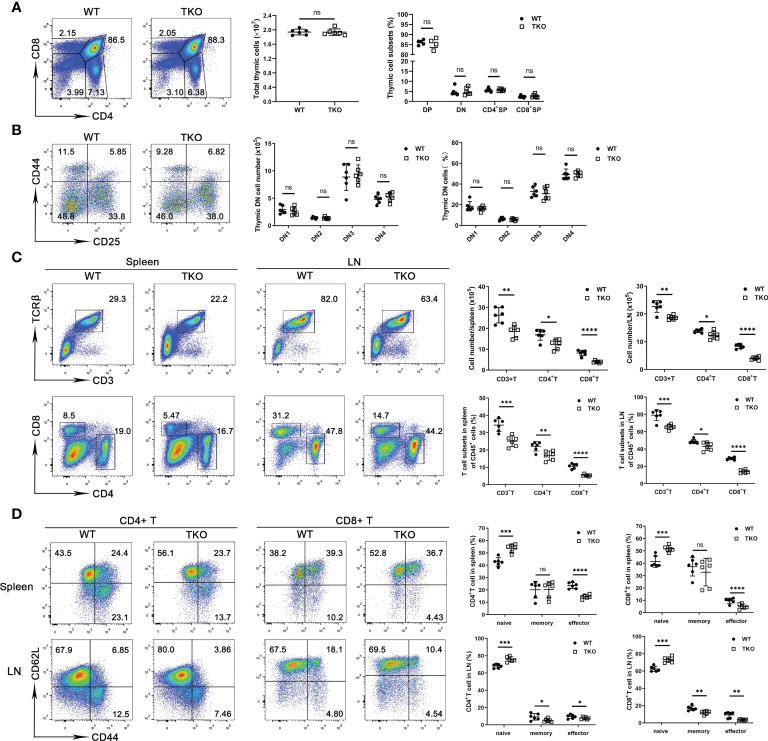
*Ccdc134* deficiency perturbs T cell homeostasis and activation. **(A, B)** Thymocytes from 4-week-old *CD4-cre^-^Ccdc134 ^fl/fl^* (WT) and *CD4-cre^+^Ccdc134 ^fl/fl^* (TKO) mice were analyzed by flow cytometry for CD4, CD8, CD44, and CD25 expression. **(A)** Surface staining of CD4 and CD8 on WT and *Ccdc134* TKO thymocytes. Left, representative plots of CD4^+^ and CD8^+^ thymocytes in the lymphocyte gating are shown. Right, quantification of the total numbers and mean frequencies of DN, DP, CD4^+^ SP and CD8^+^ SP thymocyte subpopulations. **(B)** Representative plots and quantification of CD44 and CD25 expression on DN thymocytes from WT and TKO mice. DN1: CD44^+^CD25^-^, DN2: CD44^+^CD25^+^, DN3: CD44^-^CD25^-^, DN4: CD44^-^CD25^+^. **(C)** Representative plots and quantification of TCRβ^+^CD3^+^, CD4^+^ and CD8^+^ cells gated on CD45^+^ cells from spleen and lymph node of WT and TKO mice. **(D)** Representative plots and quantification of naïve (CD44^lo^CD62L^hi^), central memory (memory, CD44^hi^CD62L^hi^) and effector memory (effector, CD44^hi^CD62L^lo^) CD4^+^ T cells and CD8^+^ T cells in the spleen and lymph node of WT and TKO mice. Data are representative of three independent experiments. The numbers in or adjacent to outlined areas (or in quadrants) indicate percentages. Each symbol represents an individual mouse. Summary graphs are presented as mean ± SEM (n = 6 mice per group) with p values determined by two-tailed Student’s t test. * *p* < 0.05; ** *p* < 0.01; *** *p* < 0.001; **** *p* < 0.0001; ns, not significant.

We next analyzed thymocyte development in 4-week-old mice with *Ccdc134* deficiency in T cells. No significant difference in absolute cell numbers or thymocyte subsets were observed in *Ccdc134* TKO mice compared with WT littermate controls (**Figure 1A**). Moreover, the proportions and numbers of CD4^–^CD8^–^ DN thymocytes within the DN1-DN4 [DN1 (CD44^+^CD25^−^), DN2 (CD44^+^CD25^+^), DN3 (CD44^−^CD25^+^), and DN4 (CD44^−^CD25^−^)] developmental stages were not affected in *Ccdc134* TKO mice (**Figure 1B**). To investigate the role of CCDC134 in homeostatic regulation of peripheral T cells, we analyzed the frequency of T cell subsets in the peripheral lymphoid organs of young adult mice at 8 weeks of age. *Ccdc134* deficiency in T cells resulted in fewer peripheral mature CD4^+^ T cells and especially CD8^+^ T cells in the spleen and lymph node (**Figure 1C**). Naïve (CD44^low^CD62L^high^) T cells differentiate into effector (CD44^hi^CD62L^low^) or central memory (CD44^hi^CD62L^hi^) T cells with self-renewal ability in response to antigen stimulation, and then migrate to lymphoid tissue for homeostatic proliferation. As shown in **Figure 1D**, both splenic and LN CD4^+^ and CD8^+^ T cells from *Ccdc134* TKO mice contained higher frequencies of naïve cells but fewer effector or memory cells than littermate control mice. Similar results were observed in mice with Lck-driven T cell-specific deficiency of *Ccdc134*. These results suggest that CCDC134 plays an important role in regulating the homeostasis of peripheral T cells, particularly CD8^+^ T cells.

### *Ccdc134*-deficient T cells exhibit decreased responses to TCR stimulation *in vitro*

**Figure 2:**
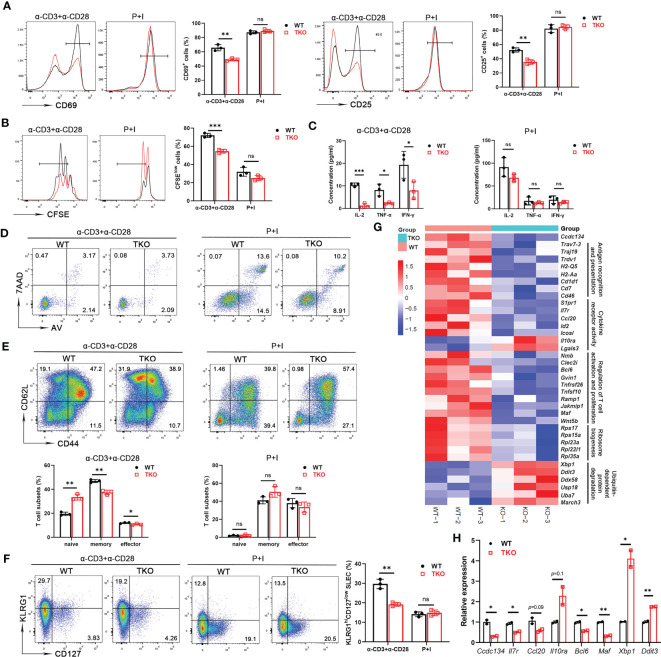
*Ccdc134* deficiency attenuates T cell responses *in vitro*. Naïve CD8^+^ splenic T cells from the spleens of 8-week-old *CD4-cre^-^Ccdc134 ^fl/fl^* (WT) and *CD4-cre^+^Ccdc134 ^fl/fl^* (TKO) mice were stimulated with plate-bound anti-CD3 (2μg/ml) and anti-CD28 (1μg/ml) antibodies, or PMA and ionomycin (P+I) for different time points. **(A)** Representative plots and quantification of CD25 and CD69 expression in CD8^+^ splenic T cells stimulated for 24 h were analyzed by FACS. **(B)** Representative plots and quantification of cell proliferation in CD8^+^ splenic T cells stimulated for 48 h were measured by CFSE assay. **(C)** Concentrations of IL-2, TNFα, and IFNγ in culture supernatants of CD8^+^ splenic T cells stimulated for 48 h were measured by ELISA. **(D)** Flow cytometric analysis of apoptotic CD8^+^ T cells (Annexin V^+^) stimulated for 48 h. **(E)** Representative plots and quantification of flow cytometric analysis of naïve, central memory and effector memory populations in CD8^+^ T cells from spleens of WT and *Ccdc134* TKO mice after 48 h of stimulation. **(F)** Representative plots and quantification of flow cytometric analysis of short-lived effector cells (KLRG1^hi^CD127^low^ SLEC) or memory precursor effector cells (KLRG1^low^CD127^hi^ MPEC) of CD8^+^ T cells from WT and TKO mice stimulated for 48 h. **(G)** Heat map showing gene expression changes identified from RNA sequencing analysis of total T cells from WT and *Ccdc134* TKO mice. Genes are organized as related to the categories shown to the right. **(H)** The qRT-PCR analysis of the indicated genes in total CD8^+^ T cells. Relative expression indicates the change in target gene expression relative to *Gapdh* expression in the same sample compared with WT control. The representative results and quantification were shown, and the experiments were repeated at least three times. Each symbol represents an individual mouse. Summary graphs are presented as mean ± SEM (n = 3 mice per group) with p values determined by two-tailed Student’s t test. * *p* < 0.05; ** *p* < 0.01; *** *p* < 0.001; ns, not significant.

Homeostatic generation of effector T cells involves TCR signaling in response to various activation signals. The reduced frequency of effector or memory T cells in *Ccdc134* TKO mice prompted us to examine the role of CCDC134 in regulating T cell activation. Naïve CD8^+^ T cells were stimulated *in vitro* with agonistic antibodies for TCR (anti-CD3ϵ) and CD28 (anti-CD28), or phorbol ester (PMA) and calcium ionophore (ionomycin), which bypass the proximal TCR signaling pathway, and activation and proliferation were measured. Following TCR crosslinking with anti-CD3ϵ and anti-CD28 antibodies, *Ccdc134*-deficient T cells were attenuated in early activation as revealed by significant downregulation of the early and transient T-cell activation markers CD69 and CD25 (**Figure 2A**) and underwent fewer cell divisions than WT CD8^+^ T cells in a CFSE assay (**Figure 2B**). Parallel ELISA revealed a profound reduction of IL-2, TNF-α, and IFN-γ in the CD8^+^ T cells of *Ccdc134* TKO mice (**Figure 2C**). However, no differences were observed when *Ccdc134*-deficient T cells were treated with PMA and ionomycin (**Figures 2A–C**), suggesting that *Ccdc134* deficiency specifically decreases TCR-mediated T cell responses *in vitro*.

To exclude the possibility that inhibition of T cell proliferation was due to increased apoptosis, we assessed CD8^+^ T cell survival. Splenic CD8^+^ T cells isolated from *Ccdc134* TKO and WT mice were either left unstimulated or stimulated with anti-CD3 and anti-CD28 antibodies or PMA and ionomycin overnight, followed by Annexin V and 7-AAD staining. We found that *Ccdc134* deficiency had no effect on apoptosis in T cells stimulated with antibodies or PMA and ionomycin (**Figure 2D**).

Since the central memory CD8^+^ T cells can further differentiate into effector memory CD8^+^ T cells that can then give rise to terminally differentiated effector T cells. As shown in **Figure 2E**, splenic *Ccdc134* TKO CD8^+^ T cells had lower frequencies of the central memory population than WT CD8^+^ T cells in the early stage of stimulation. We further examined the populations of short-lived effector cells (KLRG1^hi^CD127^low^, SLECs) or memory precursor effector cells (KLRG1^low^CD127^hi^, MPECs) differentiated from naïve CD8^+^ T cells during early activation. The frequency of SLECs from *Ccdc134* TKO CD8^+^ T cells was significantly reduced, while the frequency of MPEC cells was not significantly changed following antibody stimulation (**Figure 2F**).

RNA sequencing further revealed that *Ccdc134* TKO T cells had downregulated expression of genes signatures associated with T cell antigen recognition, activation and proliferation, and cytokine receptor activity, as well as upregulated expression of genes related to ubiquitin-dependent protein degradation (**Figure 2G**). We next performed quantitative qRT-PCR analysis using WT and *Ccdc134* TKO CD8^+^ T cells. Relative to WT CD8^+^ T cells, *Ccdc134* TKO CD8^+^ T cells displayed down-regulated expression of cytokine *Ccl20*, cytokine receptor *Il7r*, and T cell effector programming regulators *Bcl6* and *Maf*, and up-regulation of inhibitory cytokine receptor *Il10ra*, and unfolded protein response regulators *Xbp1* and *Ddit3* (**Figure 2H**). Together, these results suggest that *Ccdc134* deficiency affects T cell differentiation after early activation and confirmed that *Ccdc134*-deficient T cells are hyporesponsive to TCR signaling stimulation.

Since CCDC134 is also a secreted protein, we next examined if exogenous recombinant mouse CCDC134 protein might rescue the inhibitory effect of *Ccdc134* deficiency on CD8^+^ T cell activation and differentiation. As shown in **Supplemental Figures 2A, B**, exogenous recombinant mouse CCDC134 protein at both 10 ng/ml and 100ng/ml significantly promoted CD8^+^ T cell activation and proliferation, such as upregulation of surface CD25 and CD44 expression, which was similar to previous results (17). However, we found that exogenous recombinant mouse CCDC134 protein did not rescue expression of CD25 and CD44, or the proliferative capacity of CD8^+^ T cells, which is defective in CCDC134. This suggests a cell-intrinsic role for CCDC134 in maintaining peripheral CD8^+^ T cell homeostasis and activation *in vitro*.

### *Ccdc134* deficiency decreases antigen-induced T cell responses *in vivo*

**Figure 3:**
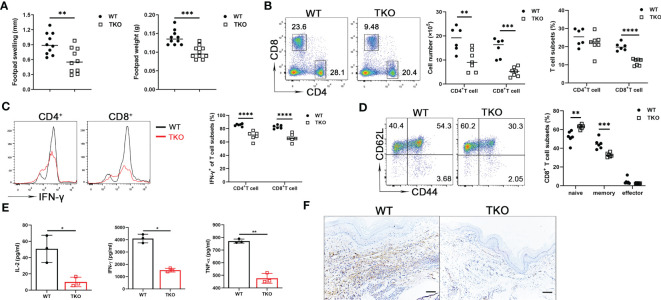
Decreased T cell response *in vivo* in *Ccdc134*-deficient mice with mBSA-induced DTH. Eight-week-old *Lck-cre^-^Ccdc134 ^fl/fl^* (WT) and *Lck-cre^+^Ccdc134 ^fl/fl^* (TKO) mice (n=10 per group) were sensitized subcutaneously with mBSA in CFA and challenged with mBSA one week later. **(A)** Footpad swelling and weight of WT and *Ccdc134* TKO 24 h after mBSA challenge. **(B)** Flow cytometric analysis of CD4^+^ and CD8^+^ T cells from popliteal draining lymph nodes (DLN) of mBSA-challenged WT and *Ccdc134* TKO mice. **(C)** Flow cytometric analysis of IFN-γ^+^CD4^+^ and IFN-γ^+^CD8^+^ T cells in the DLN. **(D)** Flow cytometric analysis of naïve (CD44^lo^CD62L^hi^), central memory (memory, CD44^hi^CD62L^hi^) and effector memory (effector, CD44^hi^CD62L^lo^) populations (gated CD8^+^). Data are presented as representative plots (left) and summary graphs (right). **(E)** Popliteal DLN cells were stimulated with anti-CD3 with anti-CD28 antibodies for 48 h and subjected to ELISA analyses for IL-2, IFN-γ and TNF-α. **(F)** Immunohistochemistry staining of the footpads from mBSA-challenged WT and *Ccdc134* TKO mice using rabbit anti-mouse CD3ϵ antibody. Scale bar = 100μm. Data are representative of two independent experiments with six or ten mice per group. Summary graphs are presented as mean ± SEM (n = 6 mice per group) with p values determined by two-tailed Student’s t test. * *p* < 0.05; ** *p* < 0.01; *** *p* < 0.001; **** *p* < 0.0001.

As a first approach to determine the *in vivo* effects of *Ccdc134* deficiency, we examined T cell responsiveness in a DTH model where immune response is primarily T cell-mediated. Mice were sensitized intradermally with mBSA in CFA in both hind footpads and challenged with the same antigen in one footpad one week later. Footpad swelling and footpad weight were measured. Compared to WT mice, *Ccdc134* TKO mice had reduced footpad swelling and weight (**Figure 3A**). Single-cell suspensions from draining popliteal lymph nodes were stimulated with anti-CD3 and anti-CD28 antibodies. Consistent with reduced swelling, TKO mice had a substantial reduction in the number and frequency of CD4^+^ and CD8^+^ T cells in draining popliteal lymph nodes (**Figure 3B**), and a reduced percentage of IFN-γ-producing CD4^+^ and CD8^+^ T cells (**Figure 3C**). Moreover, the *Ccdc134* TKO mice had lower frequencies of central memory CD8^+^ T cells than WT controls (**Figure 3D**). *Ccdc134*-deficient T cells also exhibited significantly decreased IL-2, IFN-γ, and TNF-α production following antibody stimulation for 48 h (**Figure 3E**). In addition, CD3^+^ T cell infiltration was markedly decreased in the footpad of *Ccdc134* TKO mice compared to WT control (**Figure 3F**), indicating that *Ccdc134*-deficient T cells expanded less efficiently and/or responded less vigorously *in vivo* than control T cells.

**Figure 4:**
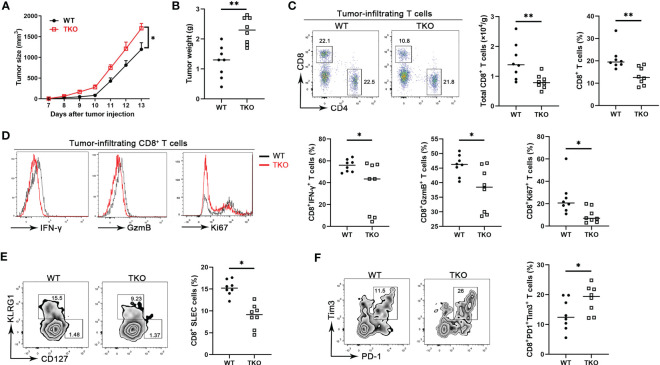
*Ccdc134* deficiency attenuates T cell anti-tumor immunity. The *Lck-cre^-^Ccdc134 ^fl/fl^* (WT) and *Lck-cre^+^Ccdc134 ^fl/fl^* (TKO) mice were injected with 2×10^5^ B16-OVA cells subcutaneously on day 0 (n = 8 per group). **(A)** Tumor size was monitored from day 7 and calculated as ½(ab^2^), where a is the length and b is the width. **(B)** B16-OVA tumor weight in WT and *Ccdc134* TKO mice on day 14 is shown. **(C)** The tumor-infiltrating cells isolated from tumor on day 14 were stained with anti-CD4 and CD8α antibodies. The absolute cell number and percentage of CD4^+^ and CD8^+^ T cells (gated CD45^+^) cells are shown. **(D)** Flow cytometric analysis of effector (IFN-γ^+^ or granzyme B^+^) and proliferative (Ki67^+^) CD8^+^ T cells, expressed as a percentage of total CD8^+^ T cells in the tumor. **(E)** Flow cytometric analysis of tumor-infiltrating SLEC or MPEC CD8^+^ T cells. **(F)** Flow cytometric analysis of tumor-infiltrating exhausted (PD1^+^Tim3^+^) CD8^+^ T cells. Data are presented as representative plots of three independent experiments with eight mice per group (left). Summary graphs are presented as mean ± SEM with *p* values determined by two-way analysis of variance (ANOVA) with Bonferroni correction **(A)** and two-tailed Student’s t test (B-F, right). * *p* < 0.05; ** *p* < 0.01.

CD8^+^ T cells are central components for cancer immunity (20), and our finding that *Ccdc134* deficiency inhibits CD8^+^ T cell activation suggested a potential role of CCDC134 in regulating antitumor immunity. To investigate the role of CCDC134 in T cell-mediated anti-tumor immunity, we assessed *Ccdc134* TKO mice in the B16-OVA murine melanoma model. Compared to WT mice, *Ccdc134* TKO mice had significantly increased tumor progression and tumor size (**Figures 4A, B**). This was coupled with decreased CD8^+^ T cell number and frequency, but not CD4^+^ T cells, in tumors (**Figure 4C**). More importantly, effector CD8^+^ T cells producing IFN-γ and granzyme B were significantly reduced in tumors and draining lymph nodes from *Ccdc134* TKO mice, relative to WT mice, and *Ccdc134* TKO CD8^+^ T cells expressed lower levels of Ki67 than WT controls in the tumor microenvironment (**Figure 4D**). The frequency of SLECs from *Ccdc134* TKO CD8^+^ T cells was significantly reduced, while the frequency of MPEC cells was not significantly changed (**Figure 4E**). In addition, the frequency of exhausted PD-1^+^Tim3^+^CD8^+^ T cells was significantly increased in tumors from *Ccdc134* TKO mice (**Figure 4F**). It has been reported that CD8^+^ T cells undergo altered effector differentiation that culminates an exhausted state defined by defective cytotoxicity, reduced pro-inflammatory cytokine production, and induction of the immunosuppressive cytokine (21). We speculate that the increased frequency of exhausted CD8^+^ T cells may lead to attenuated effector function and failure to control tumor progression in *Ccdc134* TKO mice. These data show that CCDC134 plays a key role in anti-tumor immunity by regulating T cell responses.

### *Ccdc134*-deficient T cells exhibit accelerated surface TCR downmodulation upon activation

**Figure 5:**
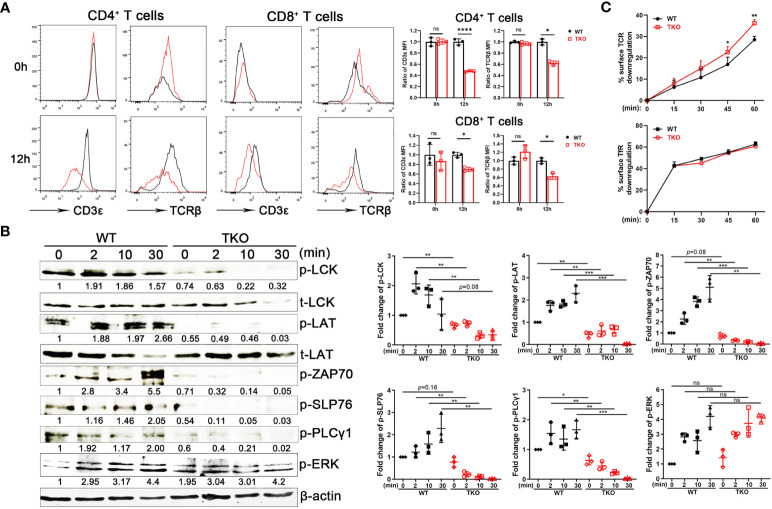
Impaired TCR signaling in *Ccdc134*-deficient T cells. **(A)** TCR downmodulation after TCR stimulation. Naïve CD4^+^ and CD8^+^ T cells from spleens of *Lck-cre^-^Ccdc134 ^fl/fl^* (WT) and *Lck-cre^+^Ccdc134 ^fl/fl^* (TKO) mice were stimulated with plate-bound anti-CD3ϵ/CD28 mAbs, and cell-surface CD3ϵ and TCRβ levels were examined at the indicated time points by flow cytometry. Representative histograms (black line for WT and red line for TKO) and quantification were shown. **(B)** Naïve CD4^+^ T cells (2×10^6^ cells per condition) from spleens of WT and *Ccdc134* TKO mice were activated with plate-bound anti-CD3ϵ/CD28 mAbs at 37°C for the indicated times. Cells were lysed and 15 μg of protein cell lysates were subjected to immunoblot analysis to detect phosphorylation of molecules involved in T cell activation. The integrated volumes of appropriately sized bands were quantified using ImageJ software. The protein content was normalized to total LCK (for phosphor-Lck, p-LCK), t-LAT (for p-LAT), and β-actin (for p-ZAP70, p-SLP76, p-PLCγ1 and p-ERK). Representative images of three independent experiments and quantification are presented. **(C)** Naïve CD4^+^ T cells (5×10^5^ cells per condition) from spleens of WT and *Ccdc134* TKO mice were treated with Dynabeads mouse T-activator CD3ϵ/CD28 for 30 min on ice. Surface TCR and TfR were quantified. Each symbol represents an individual mouse, and the experiments were repeated three times. Summary graphs are presented as mean ± SEM (n = 3 mice per group) with *p* values determined by two-tailed Student’s t test. * *p* < 0.05; ** *p* < 0.01; *** *p* < 0.001; **** *p* < 0.0001; ns, not significant.

To explore the mechanism of hyporesponsiveness to TCR-CD3-mediated signaling, we quantified cell surface TCRβ and CD3ϵ expression on splenic T cells from *Ccdc134* TKO and WT mice. There were no significant differences at steady state, however *Ccdc134* TKO T cells expressed lower levels of surface TCRβ and CD3ϵ following stimulation with plate-bound anti-CD3/CD28, relative to wild-type T cells (**Figure 5A**). Engagement of the TCR-CD3 complex results in the sequential activation of cytoplasmic LCK and ZAP-70 protein tyrosine kinases (PTKs) that promote tyrosine phosphorylation of not only the PTKs, but also the CD3ζ receptor subunits, the SLP-76 adaptor, and PLCγ1 effector enzymes as well to facilitate increased free cytoplasmic calcium, regulate actin reorganization, and downstream activation of NF-κB signaling (22). TCR crosslinking of *Ccdc134* TKO CD4^+^ T cells resulted in decreased phosphorylation of TCR proximal signaling components, including ZAP70, LCK, SLP76 and PLCγ1. Meanwhile, no significant difference in phosphorylation of ERK1/2 were found in *Ccdc134*-deficient naïve CD4^+^ T cells (**Figure 5B**). All above data suggest that the decrease of TCR signaling due to *Ccdc134* defect might inhibit T cell activation.

Given that activated T cells down-regulate cell surface TCR-CD3, a process that rapidly desensitizes the cell (5), we next analyzed the kinetics of surface TCR downmodulation. Under homeostatic conditions, surface TCR-CD3 complexes are continuously internalized and recycled back to the plasma membrane. Following antigen stimulation, surface TCRs undergo downmodulation and are targeted for intracellular degradation through lysosomes and proteasomes to attenuate TCR signaling (23). Stimulation of WT naïve CD4^+^ T cells with mouse T-activator CD3/CD28 Dynabeads resulted in downregulation of approximately 30% of surface TCRs within 15 to 60 min of receptor activation (**Figure 5C**). In contrast, *Ccdc134* TKO CD4^+^ T cells exhibited accelerated TCR downmodulation. Internalization of transferrin receptor (TfR) in CD4^+^ T cells was not affected (**Figure 5C**). These results suggest that the reduced TCR signaling observed specifically in *Ccdc134*-deficient CD4^+^ T cells may be due to accelerated surface TCR downregulation.

### CCDC134 interacts with multiple proteins involved in receptor synthesis and endocytosis

**Figure 6:**
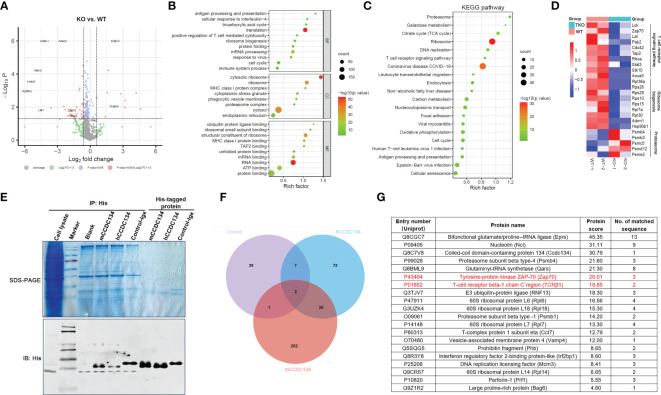
Identification of interacting partners of CCDC134. **(A-D)** The CD8^+^ T cells *from CD4-cre^-^Ccdc134 ^fl/fl^* (WT) and *CD4-cre^+^Ccdc134 ^fl/fl^* (TKO) mice were isolated and stimulated with antibodies for 16 h, and whole cell lysates were extracted for LC-MS/MS analysis. **(A)** Volcano plot of all proteins identified by LC-MS/MS analysis. The abscissa is the fold change (FC) in protein concentration and the ordinate is the statistical significance. Red dots represent the differentially expressed proteins between *Ccdc134* TKO and WT CD8^+^ T cells with significant differences, FC > 1.5 or ≤ 0.667 and *P* Value < 0.05; grey dots are proteins without significant change. Data are presented as representative plot of two independent experiments. **(B)** Functional enrichment of differentially expressed proteins between *Ccdc134* TKO and WT CD8^+^ T cells. The circle size indicates the number of differentially expressed proteins, and the circle color indicates the enrichment significance of the *p* value. BP, biological processes; CC, cellular components; MF, molecular functions. **(C, D)** KEGG pathways enriched by differentially expressed proteins **(C)** and heatmap of differentially expressed proteins **(D)** between *Ccdc134* TKO and WT CD8^+^ T cells. Each column is one sample and color scale represent the intensity scale as calculated by log2 transformation. **(E)** Whole cell lysates from mice splenic T cells were used to pull down with recombinant eukaryotic His-tagged mCCDC134 or hCCDC134 proteins, or the control protein. The complexes were separated by SDS-PAGE and detected by immunoblot analysis. **(F)** Venn diagram of interacting partners among mCCDC134, hCCDC134, and control protein. **(G)** Twenty potential interacting proteins for CCDC134 are shown. Data are presented as representative plots of two independent experiments.

We next sought to gain insight into the role of CCDC134 in the synthesis, trafficking, and turnover of TCR-CD3 complexes by identifying binding partners of CCDC134 in T cells. CD8^+^ T cells from *Ccdc134* TKO and WT mice were isolated and stimulated with antibodies for 16 h, and whole cell lysates were extracted for LC-mass spectrometry (MS)/MS analysis. We identified and quantified 939 proteins, among which 11 were upregulated with a fold change >1.5 (p < 0.05) and 40 were downregulated with a fold change < 1/1.5 (p < 0.05). Volcano plot analysis was conducted to visualize differentially expressed proteins, such as Zap70 and Hspa8, but not TCR-CD3 complexes in *Ccdc134* TKO and WT CD8^+^ T cells, perhaps it is because little membrane protein was extracted and detected (**Figure 6A**).

To further investigate functional classifications and pathways associated with these differentially expressed proteins, we performed GO and KEGG pathway enrichment analysis. As shown in **Figure 6B**, differentially expressed proteins in the *Ccdc134* TKO group were enriched for translation and mRNA processing using analysis of biological process category. Cellular component category indicated they were mainly located in the cytosol and ribosome, and molecular function analysis showed enrichment for proteins involved in the structural constituent of ribosomes and RNA binding. Further KEGG pathway analysis revealed enrichment for several vital pathways, including proteasome and ribosome (**Figure 6C**). As expected, we found that the TCR signaling pathway was down-regulated in *Ccdc134* TKO CD8^+^ T cells compared with WT controls. This included the T cell function-related proteins Lck, Zap70 and Lat as important kinases downstream of TCR-CD3 signaling, and T cell activation related proteins, such as Stat3 and Hsp90b1, as well as transporter associated with antigen processing (Tap2), which is associated with T cell antigen-specific recognition (**Figure 6D**), suggesting that *Ccdc134* deficiency may affect the TCR-CD3 signaling pathway. In addition, several metabolism-related molecules were significantly downregulated in *Ccdc134*-deficient CD8^+^ T cells, such as ribosomal function-related proteins (**Figure 6D**). As metabolic reprogramming is a hallmark of T cell activation and is required for the function of effector T cells (24), the finding indicates that *Ccdc134* deficiency might diminish the metabolic level of CD8^+^ T cells and in turn lead to impaired cell activation.

We further performed a pull-down assay in lysates from splenic T cells with recombinant eukaryotic His-tagged mCCDC134 or hCCDC134 proteins and analyzed the products by LC-MS/MS (**Figure 6E**). We conducted Venn analyses to identify key proteins that interacted with mCCDC134 or hCCDC134. Consistent with our prior results linking CCDC134 with TCR-CD3 components, we identified twenty novel interacting partners for CCDC134 with established roles in the synthesis and turnover of TCR-CD3 complex, including TCRβ1 and Zap70 (**Figures 6F, G**). CCDC134 was also bound to some proteins related to the ubiquitin-proteasome degradation pathway including proteasome subunit beta type-4 (Psmb4), E3 ubiquitin-protein ligase (RNF13), and BCL2-associated athanogene 6 (Bag6), which is involved in the polyubiquitin-dependent proteasome mediated degradation pathway. In addition, Perforin-1(Prf1), as well as 60S ribosomal proteins L6, L7, L14, and L18, were also identified as interacting partners of CCDC134 (**Figure 6G**). Prf1 serves as part of the transmembrane pore which is essential for cytotoxic lymphocyte function and immune regulation in the host (25), suggesting that *Ccdc134* deficiency might also affect lytic molecules production which might contribute to reduced cytotoxicity. Taken together, our functional data supported by our proteomic results and enrichment analysis, suggest that CCDC134 plays a significant role in the synthesis and turnover of the TCR-CD3 complex in the cytoplasm, likely *via* ubiquitin-proteasome degradation pathway, as well as in the dynamics and equilibrium of cell-surface TCR-CD3 *via* endocytosis.

### CCDC134 binds to CD3ϵ and promotes CD3ϵ ubiquitination

**Figure 7:**
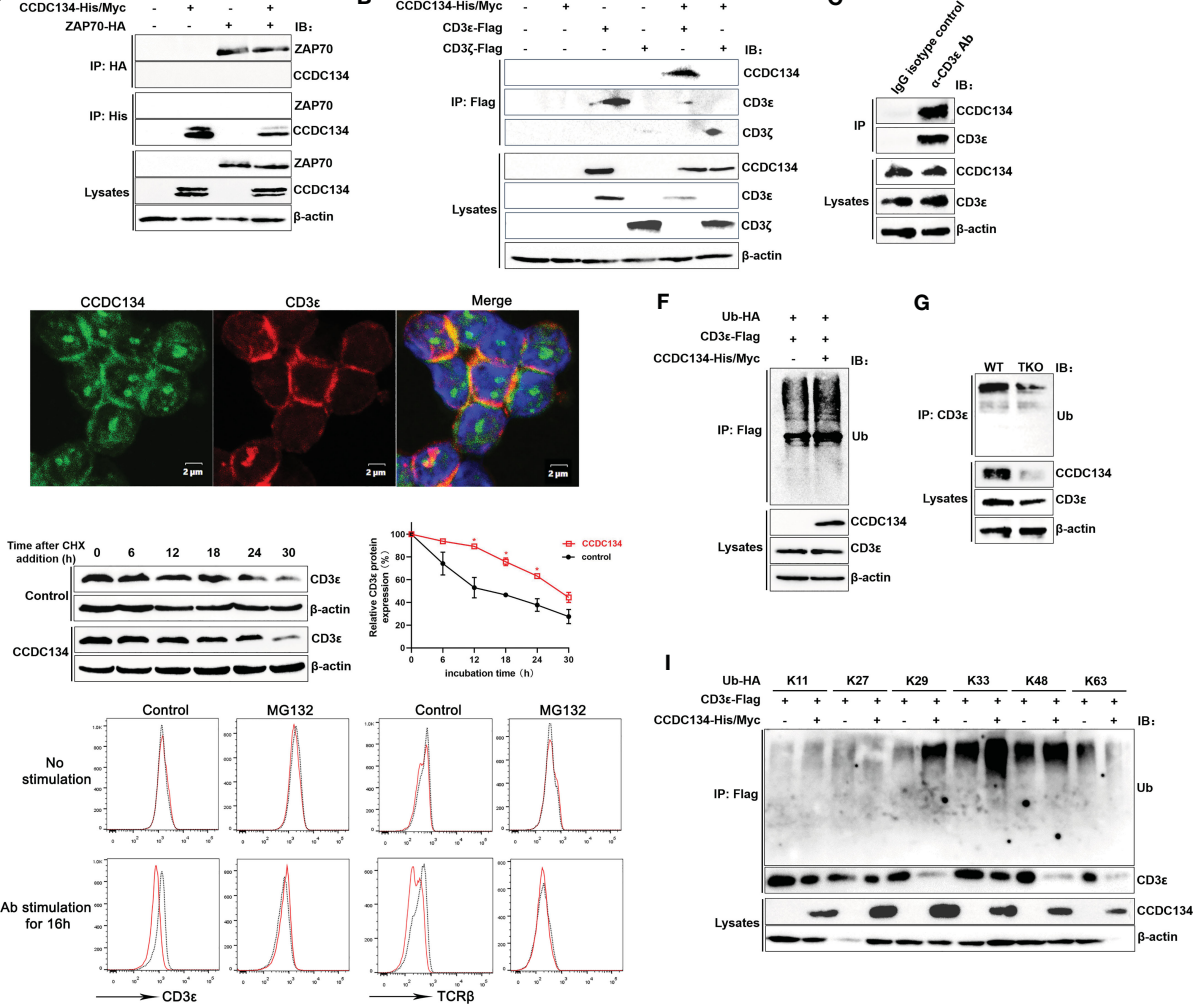
CCDC134 binds to CD3ϵ and promotes CD3ϵ ubiquitination. **(A)** Immunoblot analysis of CCDC134 and ZAP70 interaction. HEK293T cells were transfected with CCDC134-His/Myc and ZAP70-HA. The lysates were subjected to immunoprecipitation using an anti-HA or anti-His antibody and blots were probed with anti-HA or anti-His antibodies. **(B)** Immunoblot analysis of CCDC134 and CD3ϵ or CD3ζ interaction. HEK293T cells were transfected with CCDC134-His/Myc and CD3ϵ-Flag or CD3ζ-Flag. The lysates were subjected to immunoprecipitation using an anti-Flag antibody and the blots were probed with anti-His or anti-Flag antibodies. Below, immunoblot analysis of lysates (without immunoprecipitation) is also shown. β-actin was used as a loading control. **(C)** The interaction between endogenous CCDC134 and CD3ϵ was shown in the coimmunoprecipitation assay. The cell lysate of primary mouse CD3^+^ T cells was immunoprecipitated with rat anti-CD3ϵ antibody and rat IgG as control. **(D)** Confocal microscopy analysis of CCDC134 (green) and CD3ϵ (red) in isolated mouse CD8^+^ T cells using rabbit anti-mouse CCDC134 antibody and rat anti-mouse CD3ϵ antibody. DAPI (blue) was used for nuclear staining. Scale bar, 2 μm. **(E)** Overexpression of CCDC134 decreases the decay of CD3ϵ. HEK293T cells were transiently cotransfected with CD3ϵ-Flag and either CCDC134 or empty vector. After 36 h, cells were treated with 100 μg/ml of CHX for the indicated times. Total cell lysates were analyzed for CD3ϵ by immunoblot. The intensity of CD3ϵ relative to β-actin was quantified by densitometry using ImageJ software, and plotted against the time of cycloheximide treatment (below). This experiment was performed twice. **(F)** HEK293T cells were transfected with CD3ϵ-Flag and Ub-HA, either in the presence (+) or absence (–) of CCDC134-His/Myc. Whole cell lysates were prepared from MG132-treated (6 h) cells and subjected to immunoprecipitation with an anti-Flag antibody followed by detection of ubiquitinated CD3ϵ with anti-HA antibody. **(G)** Whole cell lysates of isolated mouse CD8^+^ T cells from *CD4-cre^-^Ccdc134 ^fl/fl^* (WT) and *CD4-cre^+^Ccdc134 ^fl/fl^* (TKO) mice were prepared and subjected to immunoprecipitation with an anti-CD3ϵ antibody followed by detection of ubiquitinated CD3ϵ with anti-ubiquitin antibody. **(H)** The isolated CD8^+^ T cells from *CD4-cre^-^Ccdc134 ^fl/fl^* (WT) and *CD4-cre^+^Ccdc134 ^fl/f^* (TKO) mice were stimulated *in vitro* using plate-bound anti-CD3ϵ (2 μg/mL) and soluble anti-CD28 (1 μg/mL) for 12 h, and then were pretreated with the proteasome inhibitor MG132 (20 μM) for 6 h before harvesting the cells. Afterward, the cell surface CD3ϵ and TCRβ were detected by FACS analysis. **(I)** HEK293T cells were transfected with CD3ϵ-Flag and Ub-HA mutants that express only the indicated lysine with all other lysines mutated to arginines either in the presence (+) or absence (–) of CCDC134-His/Myc. Whole cell lysates were prepared from MG132-treated (6 h) cells and subjected to immunoprecipitation with an anti-Flag antibody followed by detection of ubiquitinated CD3ϵ with anti-HA. Below, immunoblot analysis of lysates without immunoprecipitation is shown. β-actin was used as a loading control.

Because our MS analysis identified the TCR-CD3 complex and downstream ZAP70 as potential novel interacting partners for CCDC134, we next investigated if CCDC134 binds to TCR-CD3 complex or ZAP70. Co-immunoprecipitation of lysates from HEK293T cells co-transfected with CCDC134 and CD3ϵ, or CCDC134 and ZAP70 showed that CCDC134 interacted with CD3ϵ, but not ZAP70 (**Figures 7A, B**). Since the TCR-CD3 complex includes TCRαβ or TCRγδ, CD3ϵδ, CD3ϵγ, and CD3ζζ dimers, we used CD3ζ as a control to explore whether CCDC134 specifically binds to CD3ϵ. As shown in **Figure 7B**, CCDC134 interacts with CD3ϵ but not CD3ζ. We also performed co-immunoprecipitation assay to identify the interaction of CCDC134 and CD3ϵ in primary CD3^+^ T cells, and observed their interaction (**Figure 7C**). In addition, we made the colocalization of CCDC134 and CD3ϵ in primary CD8^+^ T cells using fluorescence confocal assay, and CCDC134 was partly co-localized with CD3ϵ, and meanwhile the nuclear localization of CCDC134 was also shown (**Figure 7D**).

To determine if the reduced cell surface TCR-CD3 level associated with CCDC134 expression is due to changes in the total amount of CD3ϵ, we assessed the role of CCDC134 on CD3ϵ protein stability by cycloheximide chase assay. HEK293T cells were transiently cotransfected with CD3ϵ and either the CCDC134 plasmid or a vector control. Cells were then treated with the translation inhibitor CHX (100 μg/mL) for different periods as indicated. As shown in **Figure 7E**, overexpression of CCDC134 resulted in a significant slow in CD3ϵ decay, indicating that CCDC134 was likely to reduce the CD3ϵ protein degradation *via* a post-transcriptional mechanism.

Degradation of TCR-CD3 complex subunits is a control mechanism that operates at different stages of the TCR life cycle, including synthesis, assembly, dynamics, and signal transduction. This not only controls steady-state levels of surface TCR-CD3 in resting T cells, but also moderates further T cell stimulation by downregulating TCR-CD3 surface expression upon antigen stimulation (4). After TCR triggering, CD3ϵ is targeted by ubiquitination before its degradation. We next examined whether the pattern of CD3ϵ ubiquitination was altered by CCDC134. CD3ϵ was immunoprecipitated from lysates of HEK293T cells that were transiently cotransfected with CD3ϵ, ubiquitin (Ub), and either the CCDC134 plasmid or a vector control. The result indicated that CCDC134 overexpression promoted CD3ϵ ubiquitination (**Figure 7F**). We also isolated primary CD8^+^ T cells from *Ccdc134* TKO and WT mice to detect CD3ϵ ubiquitination, and the result indicated that *Ccdc134* deficiency indeed inhibited CD3ϵ ubiquitination (**Figure 7G**). Since the ubiquitin/proteasome pathway is the principal cellular system for protein degradation, to investigate whether CCDC134 promoted TCR-CD3ϵ degradation through ubiquitin/proteasome pathway, we further treated mouse CD8^+^ T cells with the proteasome inhibitor MG132 and analyzed cell surface CD3ϵ and TCRβ expression. As shown in **Figure 7H**, there were no significant differences at steady state between *Ccdc134* TKO and WT control, and *Ccdc134* TKO T cells relative to WT group exhibited lower levels of surface TCRβ and CD3ϵ after activation, which was similar to previous results. However, the treatment of MG132 to inhibit proteasome activity indeed rescued TCRβ and CD3ϵ levels on the surface of *Ccdc134* TKO CD8^+^ T cells after activation, suggesting that *Ccdc134*-deficient T cells may be due to accelerated surface TCR downregulation dependent on proteasome degradation.

To further clarify the specific type of CD3ϵ polyubiquitination affected by CCDC134, CD3ϵ was overexpressed with different types of ubiquitin plasmid. CCDC134 overexpression specifically enhanced CD3ϵ ubiquitination, which was efficient for K29, K33 and K48-linked but not K11, K27 and K63-linked polyubiquitin chains (**Figure 7I**). Taken together, these results indicated that CCDC134 enhanced expression of TCR-CD3ϵ complex by promoting the accumulation of CD3ϵ protein, thus delayed TCR downmodulation.

## Discussion

In present study, we describe a previously unrecognized mechanism by which CCDC134 regulates the dynamics of surface TCRs to finetune T cell activation and effector function. It has been shown that CCDC134 expression and secretion are upregulated following T cell activation, especially in CD8^+^ T cells (17). Here we further identify that *Ccdc134* deficiency accelerates TCR downmodulation by ubiquitinating CD3ϵ and its downstream pathways following TCR activation, rendering mice refractory to T cell-mediated inflammatory and anti-tumor responses.

We have previously shown that CCDC134 as a potential member of the γc cytokine family significantly augmented the activation, proliferation, and cytotoxicity of CD8^+^ T cells *in vitro* and recombinant hCCDC134 protein exerted anti-tumor activity in a CD8^+^ T cell-dependent manner *in vivo (* 17). In the present study we generated T cell-specific *Ccdc134*-deficient mice and found that *Ccdc134* deficiency had no effect on the development of thymocytes, but reduced the number and frequency of peripheral T cells, especially CD8^+^ T cells, under homeostatic conditions. While there was no difference in the B cell compartment (data not shown), suggesting that *Ccdc134* deficiency in T cells specifically affected peripheral T cell homeostasis. RNA sequencing showed that *Ccdc134* deficiency led to downregulated expression of genes related to antigen recognition and presentation, as well as T cell activation and proliferation. It was mentioned in our previous study that immature dendritic cells were induced phenotypic maturation with upregulated expression of CCDC134 transcript (17), suggesting that CCDC134 might be involved in antigen recognition and presentation process. More importantly, our data suggest that *Ccdc134* deficiency impairs T cell activation and effector functions in a TCR-dependent manner. This impairment could not be rescued efficiently by exogenous recombinant mCCDC134 protein, suggesting a cell-intrinsic role for CCDC134 in maintaining T cell homeostasis and activation.

CCDC134 protein is composed of two evolutionary conserved domains: a N-terminal classical signal peptide sequence and a C-terminal nuclear domain. It was identified as a novel nuclear protein that participates in the p300/CBP-associated factor complex *via* hADA2a and affects its acetyltransferase activity (26). Additionally, we observed the presence of CCDC134 in cytoplasmic vesicles and endoplasmic reticulum, and the disruption of *Ccdc134* in mice led to embryonic lethality while loss-of function mutations in *CCDC134* are responsible for a severe autosomal recessive form of osteogenesis imperfecta (16, 27), suggesting the function of CCDC134 is multifaceted and complex. Other studies have reported that some cytokines, such as IL-33, serve as both a chromatin-associated nuclear factor and a novel cytokine of IL-1 family that induces type 2 immune responses through activation of ST2 receptor (28).

Our data suggest that CCDC134 regulates T cell homeostasis, activation and effector function *in vitro.* Moreover, *Ccdc134*-deficient T cells responded less vigorously to antigen stimulation and promoted tumor growth *in vivo*. This was associated with decreased IFN-γ-producing CD4^+^ and CD8^+^ T cell but not IL17A-producing Th17 cells (data not shown) in the DTH model, and reduced CD4^+^ and CD8^+^ T cell infiltration in tumor model, especially inhibition of CD8^+^ T cell effector activity and increased CD8^+^ T cell exhaustion, suggesting *Ccdc134* deficiency affects more CD8^+^ T cells than CD4^+^ T cells. The influence of the strength of TCR signals on CD4^+^ helper or CD8^+^ cytotoxic fate has been reported, such as through modulation of Lck activity and exchange of the intracellular domain between the CD4 and CD8 protein (29). Moreover, during early activation antigen-stimulated naïve CD8^+^ T cells expand and differentiate into a heterogeneous population of effector cells, including SLECs responsible for mediating pathogen clearance and secreting effector cytokines and MPECs as the effector cells that eventually give rise to long-term memory cells (30, 31). During tumor progression, exhausted CD8^+^ T cells progressively lose functional capabilities such as cytokine production, cytotoxicity and proliferative capacity, compared to effector CD8^+^ T cells, but still have the potential to form memory cells if removed from chronic antigen stimulation (32). Weak activation of CD8^+^ T cells following suboptimal TCR signaling has been linked to ribosome biogenesis, a rate-limiting factor in both cell growth and proliferation, which showed impaired production of ribosomes and a failure to maintain proliferative capacity after stimulation. Our results show that CCDC134 may also be involved in regulation of ribosome biogenesis, which is a highly regulated process involving rRNAs, ribosomal proteins, associated factors, and small nuclear RNAs (33). Most of our knowledge about T cell regulation of ribosome biogenesis and mRNA translation relates to the various environmental signals, which are provided to naïve T cells during their activation in lymphoid organs (34). These findings were consistent with the role of CCDC134 in decreasing the activation and proliferation of T cells, further reducing T cell’s response to inflammatory and tumor stress.

*Ccdc134*-deficient T cells expressed similar amounts of cell surface TCRβ and CD3ϵ as WT controls in the resting state, but *Ccdc134*-deficient T cells accelerated downmodulation of cell surface TCRβ and CD3ϵ upon TCR engagement. This was further correlated to dampened proximal TCR signaling, including activation of Lck, Zap70, SLP-76 and PLCγ1. Moreover, the protein-protein interactions we detected by mass spectrometry include components of the T cell receptor signaling pathway and proteasome degradation, suggesting that CCDC134 might play an important role in TCR-CD3 receptor down-modulation. The results of MS analysis also showed other molecules, such as Stat3 and Hsp90b1, was down-regulated in *Ccdc134*-deficient CD8^+^ T cells. Stat3 is a converging point of multiple oncogenic pathways, the activation of which in CD8^+^ T cells can significantly improve the anti-apoptosis and tumor killing ability of CD8^+^ T cells (35). And the deletion of *Stat3* in CD8^+^ T cells leads to the impairment of cytotoxic function and the decrease of T cell number to accelerate tumor growth (35). Hsp90b1 encoded gp96 is an ER protein with functions as a molecular chaperone and Ca^2+^ buffering protein, and loss of *gp96* results in CD4^+^ T-cell activation defects (36), suggesting *Ccdc134* deficiency might inhibit T cell activation *via* regulating Stat3 signaling and Hsp90b1 expression, however these conjectures need further experimental verification.

Further investigation showed that CCDC134 interacts with CD3ϵ and promotes its ubiquitination, and specifically enhanced K29, K33 and K48-linked polyubiquitin chains, which corresponded with higher levels of surface TCR. Although the K48- linked ubiquitination promoted protein proteasomal processing, while K29-linked ubiquitination promoted the accumulation and aggregation of insoluble Ub-containing protein, and K33-linked ubiquitination disturbs protein-protein interactions to abrogate protein proteasomal degradation, so *Ccdc134* deficiency might decrease expression of surface TCR-CD3ϵ complex by inhibiting the accumulation of CD3ϵ protein, thus accelerated TCR downmodulation to impair T cell activation and proliferation. It is well known that TCR downregulation limits T cell activation by TCR degradation, in which ubiquitination is a key step (37). Several E3 ubiquitin ligases, such as Cbl-b, GRAIL, and ITCH, negatively regulate TCR signaling by targeting TCR-signaling molecules for ubiquitin-dependent degradation (37). Blockade of the degradation of activated TCR is adverse for T cell function, however there is no experimental proof that 100% of the engaged TCR is rapidly degraded. It may be due to the fact that part of the antigen-triggered TCR is diverted from the degradation pathway to other compartments of the endocytic pathway, including recycling, endosomal signaling, or both. Additionally, surface expression of TCR on mature T cells is dependent on the assembly of receptor subunits into TCRs in the endoplasmic reticulum (ER) and their successful traversal of the secretory pathway to the plasma membrane. TCR subunits, such as TCRα and CD3δ that fail to exit the ER for the Golgi complex are degraded by non-lysosomal processes that have been referred to as “ER degradation” (6). Our RNA sequencing and proteomic studies revealed that *Ccdc134* deficiency upregulated ubiquitin-related molecules, including RNF13, Psmb1, and Psmb4, as well as multiple components of the endoplasmic-reticulum-associated protein degradation (ERAD) pathway, such as Ddit3 and Xbp1. ERAD functions as a transmembrane protein quality control mechanism in eukaryotic cells by recognizing misfolded or incompletely processed polypeptides by specialized factors, including p97 ATPase (VCP). This provides the driving force to eject them from the ER and return them to the cytoplasm where they are ubiquitinated and degraded by the 26S proteasome (38–40). Therefore, the reduction in cell surface CD3ϵ observed here may reflect increased ERAD activity, and further studies will be required to determine how CCDC134 impacts this pathway.

It has also reported that CCDC134 might interact with Ubiquilin 4 (UBQLN4; UBIN; A1Up) by yeast two-hybrid assay (41). UBQLN4 serves as a member of the ubiquitin-like protein family that specifically associates with misassembled transmembrane polyubiquitinated proteins in the cytoplasm for targeted degradation *via* the ubiquitin pathway (42). More importantly, UBQLN4 has been identified as a BAG6-binding factor that eliminates newly synthesized defective polypeptides (42). Accumulating evidence suggests that the substrates of both the co- and post-translational ER targeting pathways are polyubiquitinated in the aqueous cytosol in a BAG6-dependent manner when targeting fails (43). Here we identify Bag6 as a potential interacting partner of CCDC134, suggesting that CCDC134 functions as a specific regulator of TCR signaling *via* the ubiquitin-dependent protein degradation of CD3ϵ. An interesting challenge for the future will be determining how CCDC134 collaborates with chaperones/UBQLN4/BAG6/ERAD machinery to regulate degradation of TCR subunits.

Taken together, our study demonstrates an important role for CCDC134 in TCR downmodulation, especially in CD8^+^ T cells. CCDC134 interacts with CD3ϵ *via* ubiquitin modification resulting in impaired T cell activation and effector functions. These experimental data provide a framework by which cell-intrinsic CCDC134 may regulate TCR receptor systems to maintain homeostasis and, when dysregulated, contribute to disease.

## Data availability statement

The datasets presented in this study can be found in online repositories. The names of the repository/repositories and accession number(s) can be found below: PRJCA014255 (https://ngdc.cncb.ac.cn/gsub).

## Ethics statement

The animal study was reviewed and approved by Animal Care Committee of Peking University Health Science, Beijing, China (LA2016153).

## Author contributions

TZ, QS, HG and BY performed experiments with the assistance of SY, QS and JH performed the formal analysis. JH and TZ drafted and revised the manuscript. QG and XM provided resources and supervised the study. JH and XL acquired funding. All authors contributed to the article and approved the submitted version.

## Supplementary material

Supplementary Figure 1 Targeting strategy and identification for *Ccdc134* conditional knockout mice with T cell-specific deletion (TKO). **(A)** Outline of strategy used to target the *Ccdc134* locus by homologous recombination in embryonic stem cells. Closed diamond represented flippase recognition target sites between exon 6 and 7. Closed triangles represented LoxP sites placed flanking exon 3-6. **(B)** A representative gel image of the PCR products. The wild type (WT) and targeted alleles produce products of 420 and 576 bp respectively using primers p1 and p2 (p1: 5’-CCAGACAGAGGTGAGCTGCT-3’; p2, 5’-GCACCCTGAGCCAAGTTTAG-3’). **(C)** RT-PCR analysis of Ccdc134 mRNA in sorted CD4^-^CD8^-^ (N), CD4^+^ and CD8^+^ cells (P) splenocytes from WT and TKO mice. The number of mice were displayed. The numbers represent the number of different mice. neg, negative control. **(D)** Western blot analysis was performed in isolated CD4^+^ and CD8^+^ cells from spleens of WT and TKO mice using specific rabbit anti-CCDC134 antibody.

Supplementary Figure 2 Exogenous recombinant mCcdc134 protein partly rescue *Ccdc134*-deficiency induced T cell impairment. Naïve CD8^+^ splenic T cells were purified from the spleen of 8-week-old *Lck-cre^-^Ccdc134 ^fl/fl^* (WT) and *Lck-cre^+^Ccdc134 ^fl/fl^* (TKO) mice and stimulated with plate-bound anti-CD3 (2μg/ml) and anti-CD28 (1μg/ml) antibodies in the presence or absence of rhCC (1, 10, or 100 ng/mL) or rhIL2 (20 ng/mL). **(A)** At the indicated times, stimulated cells were analyzed for the expression of activation molecule CD25 and effector molecule CD44 (24 h). **(B)** Flow cytometry analysis of CFSE dilution in WT and *Ccdc134*-TKO CD8+ splenocytes stimulated with anti-CD3/anti-CD28 mAbs for 48h. Black dotted lines represent WT and red dotted lines represent *Ccdc134*-TKO. Data are representative of two independent experiments.
