# Supplementary figures and images for: CCDC134 facilitates T cell activation through the regulation of early T cell receptor signaling
Source: Front Immunol. 2023 May 10;14:1133111. doi: 10.3389/fimmu.2023.1133111 (PMC10206301; doi:10.3389/fimmu.2023.1133111)

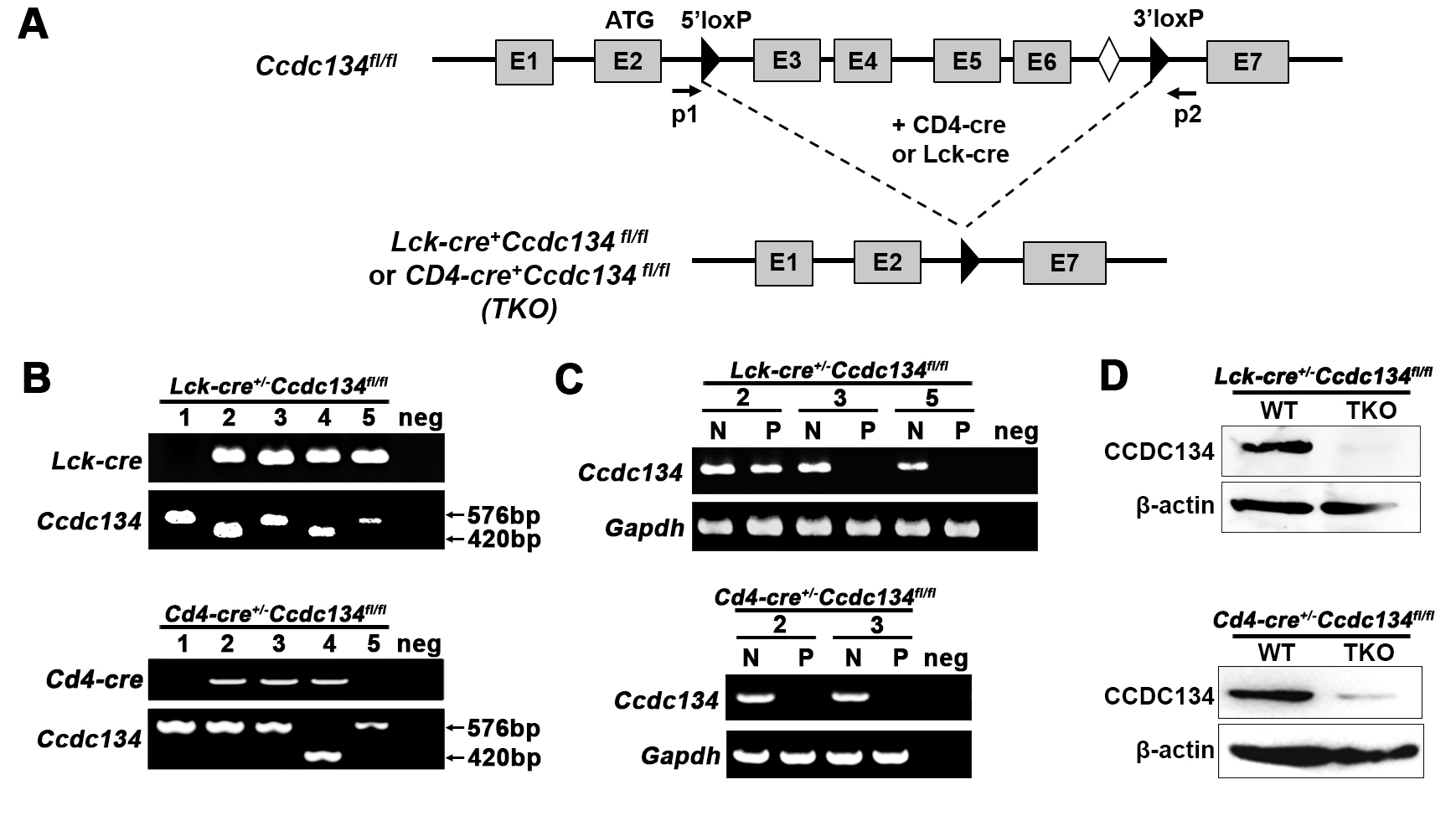

Supplement: Supplementary Figure 1 — Targeting strategy and identification for Ccdc134 conditional knockout mice with T cell-specific deletion (TKO). (A) Outline of strategy used to target the Ccdc134 locus by homologous recombination in embryonic stem cells. Closed diamond represented flippase recognition target sites between exon 6 and 7. Closed triangles represented LoxP sites placed flanking exon 3-6. (B) A representative gel image of the PCR products. The wild type (WT) and targeted alleles produce products of 420 and 576 bp respectively using primers p1 and p2 (p1: 5’-CCAGACAGAGGTGAGCTGCT-3’; p2, 5’-GCACCCTGAGCCAAGTTTAG-3’). (C) RT-PCR analysis of Ccdc134 mRNA in sorted CD4-CD8- (N), CD4+ and CD8+ cells (P) splenocytes from WT and TKO mice. The number of mice were displayed. The numbers represent the number of different mice. neg, negative control. (D) Western blot analysis was performed in isolated CD4+ and CD8+ cells from spleens of WT and TKO mice using specific rabbit anti-CCDC134 antibody. [file Image_1.jpg]

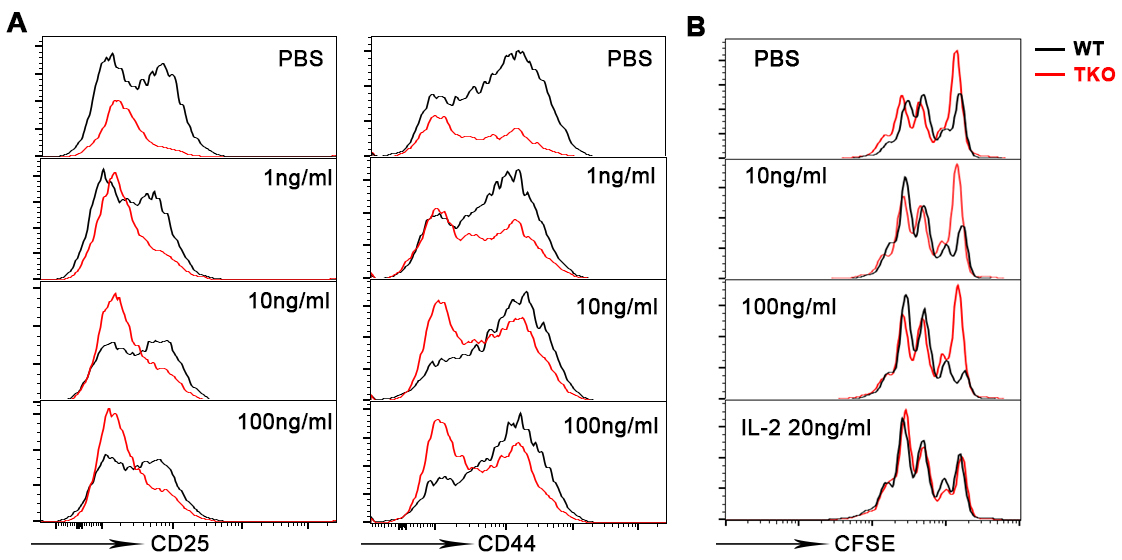

Supplement: Supplementary Figure 2 — Exogenous recombinant mCcdc134 protein partly rescue Ccdc134-deficiency induced T cell impairment. Naïve CD8+ splenic T cells were purified from the spleen of 8-week-old Lck-cre-Ccdc134 fl/fl (WT) and Lck-cre+Ccdc134 fl/fl (TKO) mice and stimulated with plate-bound anti-CD3 (2μg/ml) and anti-CD28 (1μg/ml) antibodies in the presence or absence of rhCC (1, 10, or 100 ng/mL) or rhIL2 (20 ng/mL). (A) At the indicated times, stimulated cells were analyzed for the expression of activation molecule CD25 and effector molecule CD44 (24 h). (B) Flow cytometry analysis of CFSE dilution in WT and Ccdc134-TKO CD8+ splenocytes stimulated with anti-CD3/anti-CD28 mAbs for 48h. Black dotted lines represent WT and red dotted lines represent Ccdc134-TKO. Data are representative of two independent experiments. [file Image_2.jpg]
